# An Examination of Schizotypy, Creativity, and Wellbeing in Young Populations

**DOI:** 10.3390/bs15040553

**Published:** 2025-04-19

**Authors:** Harrison E. Chapman, Sarah L. Asquith, Anna Abraham

**Affiliations:** 1 Department of Educational Psychology, Mary Frances Early College of Education, University of Georgia, Athens, GA 30602, USA; 2 Department of Psychology, Leeds Beckett University, Leeds LS1 3HE, UK; 3 Torrance Center for Creativity, Mary Frances Early College of Education, University of Georgia, Athens, GA 30602, USA

**Keywords:** schizotypal traits, psychoticism, alternate uses task, adolescence, young adulthood, life satisfaction, divergent thinking, creative thinking, mental health

## Abstract

A wide array of scholarship has revealed the somewhat paradoxical relationship between creativity and mental health. On the one hand, substantial evidence demonstrates that certain forms of mental illness are associated with enhanced creativity. On the other hand, considerable evidence also confirms that engagement in creative pursuits improves wellbeing. In this paper, we examined the associations between creative potential, the pursuit of creative hobbies, wellbeing, and schizotypy in young people aged 16–22 years. Frequentist and Bayesian approaches revealed that a higher degree of disorganized schizotypal traits was associated with greater ideational fluency and more engagement in creative hobbies, and that a higher degree of interpersonal schizotypal traits was associated with lower wellbeing. The potential drivers of this dynamic association are discussed in this paper.

## 1. Introduction

The link between creativity and psychopathology has a long history. The notion that mental illness and creative genius are inextricably linked (i.e., mad-genius hypothesis) is a persistent belief among the general public (Benedek et al., 2021). However, the nature of this relationship is, in fact, a widely debated area within the research community (Abraham, 2024; Acar et al., 2018; Taylor, 2017). Personality-based studies occupy a sizeable focus within this line of research, where subclinical psychopathological traits are examined in relation to creativity, and schizotypy is the most commonly investigated trait in this context (e.g., Abraham & Windmann, 2008; Acar & Sen, 2013; Burch et al., 2006; Mohr & Claridge, 2015).

Meehl (1962) first proposed this term to describe phenotypes of schizophrenia. The schizotypy construct consists of latent personality organizations, which may indicate a higher propensity for schizophrenia or schizophrenia-related symptoms, ranging from severe to subtle (Batey & Furnham, 2008; Lenzenweger, 2018), and may be observed in non-clinical populations (Claridge & Blakey, 2009). Schizotypy can be characterized by three primary dimensions: positive, negative, and disorganized symptoms (Mohr & Claridge, 2015). Positive symptom traits include unusual experiences, magical thinking, paranoid ideation, and suspiciousness. Negative symptom traits refer to restricted affect and social withdrawal. Cognitive disorganization traits reflect difficulty with attention, concentration, and loosening of conceptual boundaries. Self-report measures are utilized frequently to assess schizotypal traits (Mason & Claridge, 2006; Oezgen & Grant, 2018; Raine, 1991). While schizotypy scales differ in terms of factor structures and underlying concepts, comparability across measures among the positive and negative schizotypy traits is relatively high, but this is less so for the dimensions of disorganized symptoms of schizotypy (Oezgen & Grant, 2018).

Adolescence is a critical developmental period for various reflective, social, and mental health conditions (Debbané et al., 2014). Psychiatric disorders, such as schizophrenia, often manifest during early adulthood, leading current developmental approaches of psychotic disorders to focus “on high-risk or prodromal states that are temporally proximal to the onset of clinical disorder” (O’Hare et al., 2023a, p. 69). However, subclinical symptoms are often present during earlier developmental periods, such as childhood or adolescence (Debbané et al., 2014; Liu et al., 2019; O’Hare et al., 2023a). Despite this, few studies have focused on schizotypy in children (Raine et al., 2021).

Furthermore, contemporary research shows support for the cumulative risk hypothesis, which holds that larger environment risk factors (e.g., family stress, contact with child protection services, parental death, head injury) increase the likelihood of schizotypal profiles in childhood (O’Hare et al., 2023b). Childhood trauma, for instance, has also been linked to schizotypal traits, with different subdomains of trauma (e.g., physical abuse, sexual abuse, neglect) linked to different subdomains of schizotypy (e.g., perceptive aberrations, magical ideation, anhedonia) (Dizinger et al., 2022). These Adverse Childhood Experiences (ACEs, e.g., abuse, neglect, violence, bullying) increase the risk for the development of schizophrenia (Zhang et al., 2023), along with subclinical psychosis, such as schizotypy (Toutountzidis et al., 2022; Velikonja et al., 2019). The strength of the relationship between ACEs and the different dimensions of schizotypy can depend on the severity and type of traumatic experiences (e.g., sexual abuse and cognitive-perceptual features displaying a stronger association while emotional neglect shows a stronger relationship with interpersonal dimensions; Velikonja et al., 2019). A meta-analysis also supported this variation in the associations of schizotypal dimensions with different forms of ACEs, and while all forms of childhood trauma relate to schizotypy features, emotional abuse displays a stronger association (Toutountzidis et al., 2022).

The study of creativity, which refers to the generation of novel ideas that are satisfying (Abraham, 2025) or effective (Runco & Jaeger, 2012), in the context of schizotypy, typically applies the use of divergent thinking tasks to assess creative potential or performance. Divergent thinking is frequently measured using the Alternative Uses Task (AUT), a time-constrained assessment of how many different uses a person can generate for common objects (Guilford et al., 1960). The responses are evaluated in terms of a range of indices such as ideational fluency (the average number of generated uses), ideational originality (the average uniqueness of the generated uses) and peak originality (the number of highly unique uses that were generated) (Asquith et al., 2022a).

Schizotypal traits have been examined in relation to both creativity (Abraham & Windmann, 2008; Acar et al., 2018; Acar & Sen, 2013; Rominger et al., 2017) and wellbeing (Abbott et al., 2012; Tabak & Weisman de Mamani, 2013). Though higher reported levels of schizotypal traits are generally associated with lower levels of wellbeing (Abbott et al., 2012; Jacquet et al., 2020; Tabak & Weisman de Mamani, 2013), the relationship between creativity and schizotypy displays more complex associations, wherein assessing the influence of schizotypal traits by dimensions rather than a global trait offers further insight.

A meta-analysis indicated that positive symptoms of schizotypy were associated with greater creativity, whereas negative symptoms were associated with lower creativity (Acar & Sen, 2013). There is evidence to suggest that higher levels of schizotypal traits may provide a cognitive advantage in certain aspects of creative cognition, such as overcoming the constraining effects of examples and insight problem-solving (Abraham & Windmann, 2008; Karimi et al., 2007). As a whole, positive schizotypy symptoms are linked to enhanced creativity, primarily through creativity tasks measuring divergent and convergent thinking abilities (Mohr & Claridge, 2015). Schizotypy and creativity may be linked in the following ways: (a) overinclusive thinking and extending the conceptual boundaries to broaden association leads to the generation of a greater number of ideas; (b) through reduced latent inhibition; (c) regression in the service of the ego; and (d) magical thinking and unusual experiences (Acar & Sen, 2013).

Similarly, when taking symptom type into account, wellbeing also displays a complex relationship with schizotypy. Social relationships are often a key factor contributing to a person’s sense of wellbeing, but in the case of people with schizophrenia, eighty percent reported experiencing loneliness (Christensen et al., 2022). Even within non-clinical populations, people with higher levels of schizotypy often report more difficulties with social functioning (Abu-Akel et al., 2018; Fonseca-Pedrero et al., 2011). This is true for adolescents as well. For example, positive schizotypy features are associated with increased peer-relationship problems, while negative schizotypy features are related to decreased prosocial behaviors (Abu-Akel et al., 2018). Higher scores in overall schizotypy correlate with emotional difficulties, hyperactivity, and peer problems (Fonseca-Pedrero et al., 2011). Middle-age children (around the age of 11) with higher schizotypy profiles are associated with an increased likelihood of being diagnosed with a mental disorder in later adolescents (between ages 13–18; O’Hare et al., 2023a).

The specific pattern of schizotypal traits also appears to have an impact on wellbeing. After controlling for negative affect, disorganized and negative symptoms were associated with lower life satisfaction, while positive symptoms were not (Abbott et al., 2012; Fumero et al., 2018). Overall higher levels of schizotypal traits were associated with lower wellbeing; however, if the profile indicated a high level of positive traits, without concurrent higher levels of negative and disorganized traits, individuals reported similar levels of wellbeing to those with low or average schizotypal traits (Tabak & Weisman de Mamani, 2013). A cluster analysis on schizotypy identified four clusters: overall low, overall high, disorganized-interpersonal, and positive, with the overall high schizotypy cluster indicating the lowest resiliency and general maladaptation (Polner et al., 2021). This profile of high positive schizotypy with less negative/disorganized traits has led to the concept of benign or “happy” schizotypal traits (Dizinger et al., 2022) or “healthy” schizotypy (Mohr & Claridge, 2015).

Taken together, the associations among creativity, wellbeing, and schizotypy display a complex relationship that deserve further attention. Given the dearth of evidence on youth populations, there is a need to understand childhood and adolescent manifestations of schizotypal traits and their relationship with wellbeing and creativity. In the current study, using Bayesian and frequentist approaches, we investigated the relationships between schizotypal traits, creativity, and wellbeing in adolescent and young adult populations.

## 2. Materials and Methods

### 2.1. Participants

The sample (n = 76) was recruited as part of a large study previously reported in Asquith et al. (2024). Participants ranged in age from 16–22 across three age cohorts at the time of data collection. They were recruited from schools, colleges, and universities in West and North Yorkshire in the United Kingdom. Table 1 provides the socio-demographic information for the sample. The study included two phases to examine predictors of creativity and wellbeing in young people, utilizing a longitudinal, cross-sectional design. There was a two-year difference between Phase 1 and Phase 2 of the study. Three age-based cohorts were examined with the following ages during Phase 1 of the study: Cohort 1, aged 14–15 (9th grade); Cohort 2, aged 16–17 (11th grade); and Cohort 3, aged 18–20 (first year at university). Phase 2 aimed to assess the changes in predictors of creative potential and wellbeing after two years. Additionally, during Phase 2, a measure of schizotypy was added to the data collection to explore the relationship between creativity and mental illness in this population, based upon earlier findings during Phase 1 (Asquith et al., 2022b, 2024).

### 2.2. Procedure

Approval for the study was granted by the Local Research Ethics Committee at Leeds Beckett University, UK. Participants took part between November 2018 and May 2019 either in group or in individual settings in schools, colleges, or university. The session lasted a maximum of one hour. The same procedure was followed for data collection in group or individual settings. All students were given a booklet in which to record their responses. The researcher gave the students the instructions for each task using PowerPoint slides and a standard script. Students first provided sociodemographic information, then completed wellbeing, leisure, and schizotypy measures, and the creativity tasks described in the material section, along with some additional measures that are not the focus of this paper but are reported in Asquith et al. (2022b) and Asquith et al. (2024).

### 2.3. Material

#### 2.3.1. Creativity Tasks

Alternate Uses Task (AUT). Participants completed the AUT (Guilford et al., 1960), in which they were asked to come up with as many uses as possible for a common object. Three items were used (bucket, comb, and belt), and participants were given two minutes for each item and instructed to think of uses that were different from the normal use. Three measures were derived from the responses: Fluency, Overall Originality, and Peak Originality. *Fluency* was calculated as the average number of valid responses generated across the three items. *Overall Originality* was based on the relative frequency of the uses across participants (Abraham et al., 2019; Runco et al., 1987). For Overall Originality, the relative frequency was produced by taking a given use by X number of participants (e.g., 10) and dividing it by the total number of participants (e.g., 75, giving a relative frequency of 0.13). Then, the relative frequency per participant’s uses “was totaled and divided by the fluency score to give an average relative frequency, and this was subtracted from 1 so that a high score indicated high originality. The scores were averaged across the three items (Asquith et al., 2022a, p. 212). *Peak Originality* was calculated as the number of responses given by the participant that were generated by 10% or less of the sample.

Overcoming Knowledge Constraints (OKC). Participants completed a drawing task that assessed their ability to overcome knowledge constraints. They were given 5 min to imagine and draw an animal that lives on a planet just like Earth (Smith et al., 1993). Before starting to draw their creatures, participants were shown three examples that had been drawn by other participants. Drawings were scored based on whether they included three elements common to each example, four limbs, two antennae, and a tail, resulting in a range of possible scores from 0–3. The scores reflected the degree to which the participant’s drawing was constrained by the recently activated knowledge in the examples they were shown, and the ideas produced by more creative people should be less constrained by the examples (Abraham & Windmann, 2007). Scores were reversed for easier interpretation so that a higher score (OKC raw score) means a greater ability to overcome knowledge constraints.

#### 2.3.2. Wellbeing Scales

Satisfaction with Life. The five-item Satisfaction with Life Scale adapted for Children (SWLS-C) was chosen to measure life satisfaction (Gadermann et al., 2010). The scale demonstrates good convergent and discriminant validity. Participants responded on a 5-point scale (1 = *disagree a lot*, 5 = *agree a lot*) to five statements (e.g., “In most ways, my life is close to the way I would want it to be”). It provides a single life satisfaction score to measure satisfaction with life, where higher scores mean higher levels of life satisfaction (Cronbach’s α = 0.812).

Positive and Negative Experiences. The Scale of Positive and Negative Experience (SPANE) is a 12-item scale that measures positive and negative feelings (Diener et al., 2010). The SPANE has been demonstrated to have good discriminant validity within the adolescent age group (Jovanović, 2015). Participants reported how much they have experienced certain feelings (e.g., “happy”, “afraid”) over the past four weeks on a 5-point scale (1 = *very rarely or never*, 5 = *very often or always*). Responses were scored to produce sums for positive and negative affect, where higher scores reflect higher levels of positive and negative affect (Cronbach’s α = 0.825 and 0.750, respectively).

Mental Health. The 14-item Mental Health Continuum-Short Form (MHC-SF) measures emotional, social and psychological wellbeing (Keyes & Annas, 2009). Participants responded to 14 items indicating how they have been feeling during the past month, on a 6-point scale (*Never; once or twice a month; about once a week; 2 or 3 times a week; almost every day; every day*). Items include “interested in life”, “that people are basically good”, and “that you had warm and trusting relationships with others”. The data were coded to produce a total mental health score where higher scores mean higher levels of mental health (Cronbach’s α = 0.830).

Leisure Questionnaire. Participants completed a questionnaire on activities they participated in over the last month in four areas: creative hobbies, physical activity, socializing, and sedentary activities (Asquith et al., 2022a). Scores for engagement were calculated based on the number of activities and how often they engaged in them per week (less than once = 0.5, 1–2 days = 1.5, 3–4 days = 3.5, 5–6 days = 5.5, and every day = 7). Only creative hobbies were the subject of focus in the current study.

#### 2.3.3. Schizotypal Traits

Schizotypal Personality Questionnaire. The Brief Revised Updated version of the Schizotypal Personality Questionnaire (SPQ-BRU) consists of 32 questions that are summed to produce nine subscales with three or four items for each (e.g., “I sometimes feel that other people are watching me”, “I have some eccentric (odd) habits”) (Davidson et al., 2016). The questions are answered on a 5-point scale from *strongly disagree* to *strongly agree*, and higher scores mean higher levels of the trait. The scale has demonstrated good reliability and validity and supports a nine-factor single-order model relating to each of the nine sub-scales or a higher-order four-factor model: a *Cognitive Perceptual* factor, comprising Ideas of Reference, Suspiciousness, Magical Thinking and Unusual Perceptions, an *Interpersonal* factor, comprising No Close Friends and Constricted Affect, a *Disorganized* factor, comprising Eccentric Behavior and Odd Speech, and a *Total Schizotypy* score. Cronbach’s alpha for the four factors ranged from 0.807–0.881.

### 2.4. Approach to Statistical Analysis

The data were analyzed in R Studio, version 4.2.1 (R Core Team, 2022) and JASP 0.19.1 (JASP Team, 2024), and were analyzed using Frequentist and Bayesian correlations. Bayesian hypothesis testing facilitates the quantification of relative evidence for alternative and null hypotheses (Quintana & Williams, 2018; Wagenmakers et al., 2018). Unlike traditional frequentist analysis, using a Bayesian framework can help make inferences regarding the null hypothesis. Moreover, using these two inference approaches increases robustness via triangulation (Munafò & Davey Smith, 2018). For the Bayesian analysis, we selected a Bayes factor threshold of 3, as this closely corresponds to a *p*-value of 0.05 (Wetzels et al., 2015). A Bayes factor of 3 suggests that an alternative model is 3 times more favored than a null model given the data.^1^ Following the results of the Frequentist and Bayesian correlations, partial correlations were used to account for the participant’s age as a covariate on variables that met the criteria of *p* < 0.05 and BF_10_ > 3. The zero-order correlations and partial correlations were assessed for any noticeable differences in the magnitude of the relationships. If the relationships remained similar, this would suggest the initial correlation was an accurate assessment of the relationship between the two variables, even after controlling for age. However, if the partial correlation was weaker or no longer demonstrated significance, this could indicate that the initial correlation was influenced by age and may not be a direct relationship. R packages ggplot2 (Wickham et al., 2024) and ggpubr (Kassambara, 2023) were used to develop the graphs.

## 3. Results

Descriptive statistics for the creativity, schizotypy, and wellbeing variables are reported in Table 2. Frequentist and Bayesian Correlation analyses were used to examine the relationships between wellbeing, creativity, and schizotypy variables and only findings that met the threshold criteria across both types of analyses (i.e., *p* < 0.05 and BF_10_ > 3) are discussed further. Findings from the correlation analyses are reported in Table 3.

**Figure 1:**
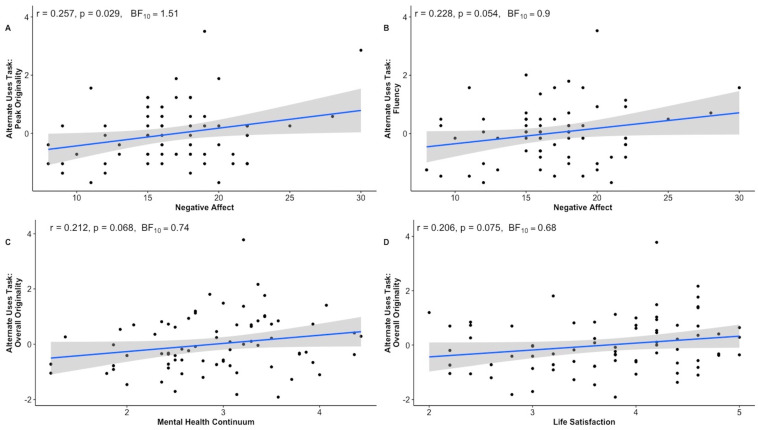
Correlation plots showing the associations between selected creativity and wellbeing measures: (**A**) Peak Originality and Negative Affect, (**B**) Ideational Fluency and Negative Affect, (**C**) Overall Originality and Mental Health, (**D**) Overall Originality and Life Satisfaction.

The findings revealed limited associations between measures of creative potential and wellbeing measures. There was partial support for the positive correlation between SPANE negative affect and AUT peak originality (i.e., significantly supported by the frequentist analysis but only anecdotally by the Bayesian analysis: *r* = 0.26, *p* = 0.029, BF_10_ = 1.51) (Figure 1). None of the other relationships between the remaining measures of creativity and wellbeing were found to be notable by either the frequentist or the Bayesian analyses (all *p* > 0.05, BF_10_ < 3). Engagement in creative hobbies was also not significantly correlated with any of wellbeing measures (all *p* > 0.05, BF_10_ < 3).

**Figure 2:**
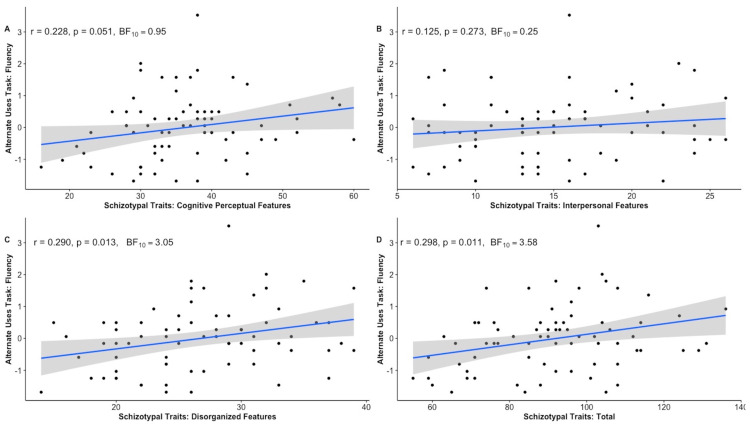
Correlation plots showing the associations between participants’ fluency scores on the Alternative Uses Task and schizotypal dimensions: (**A**) Cognitive Perceptual Factor, (**B**) Interpersonal Factor, (**C**) Disorganized Factor, (**D**) Total Schizotypy.

The analyses also revealed that specific schizotypy factors were significantly related to both creative potential and wellbeing, with positive relationships to the creativity-relevant measures and negative relationships to the wellbeing measures. Looking first at the relationship between schizotypy and creativity, the results indicated a positive relationship between total schizotypy and ideational fluency (r = 0.30, *p* = 0.011, BF_10_ = 3.58) (Figure 2). There was also partial support for the positive correlation between total schizotypy and engagement in creative hobbies (i.e., significantly supported by the frequentist analysis but only anecdotally by the Bayesian analysis; r = 0.29, *p* = 0.045, BF_10_ = 1.06). None of the other creativity measures showed notable correlations with total schizotypy (all *p* > 0.05, BF_10_ < 3). With regard to the findings in relation to schizotypy subfactors, only disorganized schizotypy was positively correlated with fluency (r = 0.29, *p* = 0.013, BF_10_ = 3.05) as well as with engagement in creative hobbies (r = 0.33, *p* = 0.004, BF_10_ = 3.77). There were no other significant associations between the schizotypy factors and creativity-relevant measures using either frequentist or Bayesian approaches.

**Figure 3:**
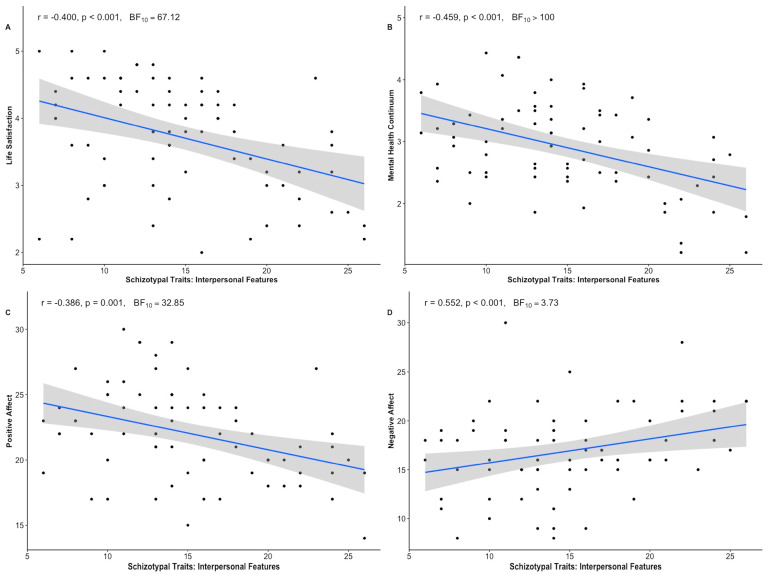
Correlation plots showing the associations between the interpersonal schizotypy factor and wellbeing measures: (**A**) Life Satisfaction, (**B**) Mental Health Continuum, (**C**) Positive Affect, (**D**) Negative Affect.

Examining the relationships between wellbeing and schizotypy, the interpersonal factor of schizotypy was associated with poor wellbeing across all four measures: life satisfaction (r = −0.40), SPANE positive affect (r = −0.39), SPANE negative affect (r = 0.30), and mental health (r = −0.45) (all *p* ≤ 0.01, all BF_10_ > 3) (Figure 3). Total schizotypy was associated with poor wellbeing across three of the four measures: life satisfaction (r = −0.39), SPANE negative affect (r = 0.49), and mental health (r = −0.41) (all *p* ≤ 0.001, all BF_10_ > 30). Disorganized features of schizotypy were positively correlated with SPANE negative affect (r = 0.30, *p* = 0.013, BF_10_ = 3.02). There was only partial support for negative correlations between the disorganized schizotypy factor and both the life satisfaction and mental health measures; they were significantly supported by the frequentist analysis, but only anecdotally by the Bayesian analysis (r = −0.26, *p* = 0.027, BF_10_ = 1.63; r = −0.27, *p* = 0.02, BF_10_ = 2.12 respectively). Similarly, the cognitive perceptual schizotypy factor was positively correlated with the SPANE negative affect (r = 0.55, *p* < 0.001, BF_10_ > 100). However, there was only partial support for negative correlations between the cognitive perceptual schizotypy factor and mental health (i.e., significantly supported by the frequentist analysis but only anecdotally by the Bayesian analysis; r = −0.27, *p* = 0.023, BF_10_ = 1.85).

Following the *p* < 0.05 and BF_10_ > 3 outlined criteria, the relationships were assessed using age as a covariate to control for potential differences across age groups. Table 4 shows the results of each partial correlation. The findings indicated support for the strength of the initial zero-order correlations. All relationships remained largely similar, with only AUT fluency and AUT overall originality displaying a somewhat stronger relationship (zero-order = 0.320, partial = 0.405). All other variables fell within ±0.040, suggesting age was not influential among the relationships of interest.

In sum, a positive relationship between schizotypy and creativity was found in relation to total schizotypy and the factor of disorganized schizotypy. Both were positively associated with AUT ideational fluency and engagement in creative hobbies. The relationship between creativity and wellbeing was highly circumscribed with partial evidence for the positive association between peak originality and negative affect. All three factors of schizotypy were negatively related to at least one measure of wellbeing, with negative affect being consistently implicated. The interpersonal factor displayed the most uniform pattern of findings as it was consistently associated with poor wellbeing across all four measures.

## 4. Discussion

The current study investigated the relationship between schizotypal traits, creativity, and wellbeing in an adolescent sample. To that end, we utilized frequentist and Bayesian approaches in correlation analyses to assess the strength of the relationship across multiple measures. Our findings demonstrated nuanced relationships between schizotypal traits, creativity, and wellbeing, partially supporting previous findings.

There was some evidence to suggest a relationship between schizotypal traits and creative potential in this sample. In particular, a high degree of disorganized features—characterized by unusual thoughts, disorganized speech patterns, and odd behaviors—was associated with greater ideational fluency and engagement in creative hobbies. Similarly, while each factor of schizotypy shared some relationship with the measures of wellbeing, the interpersonal factor—characterized by the constricted affect and absence of close friends—displayed the strongest relationship with each measure of wellbeing. Negative affect shared a positive relationship with all factors of schizotypy, suggesting all schizotypal traits increase reported negative experiences of affect.

Regarding negative affect in wellbeing, Asquith et al. (2022b) found a significant inverse relationship between negative affect and creative potential as measured by fluency and peak originality. Although, in the two-year follow-up for the current study, the findings continue to indicate that an inverse relationship is present between negative affect in wellbeing and these creativity variables, the associations did not meet the study’s a priori significance threshold for either frequentist and/or Bayesian indicators (neither threshold was reached for the ideational fluency measure; only the frequentist findings were significant for the peak originality measure). The weakening of these findings may be due to power issues due to the high dropout rate in the two-year follow-up study, which resulted in a far smaller sample size (original study: n = 391; current 2-year follow-up study: n = 76). These findings have important implications for further research in the context of creativity as well as mental health and wellbeing.

### 4.1. Schizotypal Traits and Creative Potential

In this study, only the disorganized trait factor displayed a significant relationship with both fluency and engagement in creative hobbies. Positive symptoms of schizotypy were associated with greater creative potential (Acar & Sen, 2013) and were, in part, the conception behind “happy” and “healthy” schizotypy (Dizinger et al., 2022; Mohr & Claridge, 2015). However, in our sample, cognitive perceptual features (e.g., ideas of reference, magical thinking, unusual perceptions) were not significantly correlated with ideational fluency despite showing a positive relationship. It approached significance (i.e., showed a strong trend), but did not meet the conventional thresholds for frequentist markers of significance nor Bayesian markers for even anecdotal evidence.

Disorganized schizotypal traits are generally less examined compared to the positive and negative schizotypy dimensions (Hernández et al., 2023), despite being similar to features of the “cognitive slippage” in Meehl’s (1962) original work. The disorganized traits include difficulty with organizing and expressing thoughts, speech, and behaviors (Kwapil & Barrantes-Vidal, 2015), disruptions in cognitive function (Hernández et al., 2023), and overinclusive thinking (Wang et al., 2018), which range from subtle to severe. Here, overinclusive thinking is linked with the inability to preserve conceptual boundaries, leading to “over-responsiveness to associative or irrelevant aspects of words and extraneous stimuli” (Wang et al., 2018). Hans Eysenck proposed overinclusive thinking as facilitating the link between enhanced creativity and psychoticism (Eysenck, 1995). Empirical work has shown a relationship between overinclusive thinking and creative fluency (Wang et al., 2018), which may be a result of individuals higher in disorganized traits being able to utilize remote semantic networks to make atypical connections that produce more novel ideas (Mohr & Claridge, 2015). This is in line with other work that has found schizotypal traits may provide cognitive advantages in the creative process by overcoming the constraints of the effects of examples (Abraham & Windmann, 2008) and insight problem-solving (Karimi et al., 2007).

Total schizotypal traits were also positively associated with creative fluency; however, it appears disorganized features drove the relationship followed by cognitive perceptual features, while interpersonal features displayed no meaningful relationship. These findings align with Acar and Sen’s (2013) inferences that overinclusive thinking and extension of the conceptual boundaries allow for broader associations and, thus, a greater number of ideas, and magical thinking and unusual experiences explain part of the relationship between schizotypy and creativity.

### 4.2. Schizotypal Traits and Wellbeing

Regarding wellbeing, the findings show that cognitive perceptual and disorganized schizotypal features were associated with reported increased negative affect, albeit the cognitive perceptual dimension displayed a stronger association. The interpersonal schizotypal features displayed moderate associations with all measures of wellbeing indicating lower life satisfaction, fewer experiences of positive affect, more experience of negative affect and poorer mental health. These findings suggest that while all schizotypy dimensions increase facets of negative feelings, the interpersonal traits are the driver of the negative impact of schizotypal traits on wellbeing for this young sample.

Previous studies have found similar results, with schizotypal traits being related to lower life satisfaction and higher negative affect (Abbott et al., 2012; Mohr & Claridge, 2015; Tabak & Weisman de Mamani, 2013). Fumero et al. (2018) found magical thinking to be related to several aspects of wellbeing (e.g., happiness, positive affect, personal growth) and negative schizotypal features displayed an inverse relationship with wellbeing. Similarly, a cluster of overall high schizotypal traits had lower levels of resiliency and general maladaptation (Polner et al., 2021). This would align with the link between childhood trauma, environmental risk factors, and schizotypy in early childhood and adolescence (Dizinger et al., 2022; O’Hare et al., 2023b). Furthermore, higher schizotypy features were related to various social difficulties, including peer-relationship problems and decreased prosocial behaviors (Abu-Akel et al., 2018; Fonseca-Pedrero et al., 2011). Higher schizotypal traits may be linked to various external factors (e.g., trauma, environmental factors, peer relationships) that would also impact a person’s wellbeing, which could be influenced by the various domains of schizotypy.

### 4.3. Limitations

This paper was part of a larger longitudinal study reported by Asquith et al. (2024), which followed three cohorts of adolescent participants across two different time points and assessed the predictors of creativity and wellbeing in this sample. The schizotypy questionnaire was only included during the second phase of data collection in response to the findings from the study’s first phase on the inverse relationship between creativity and wellbeing (Asquith et al., 2022b). As the schizotypy questionnaire was not used from the outset of the project in Phase 1, it is not possible to examine changes in relation to the schizotypal traits across both time points and its potential association with wellbeing and creativity. Additionally, this study focused specifically on dimensions of schizotypy and did not assess the potential for other types of subclinical or clinical mental health disorders (e.g., anxiety, depression, bipolar). Given this, this study could not examine potential relationships between other types of mental health concerns, schizotypy, wellbeing, and creativity. However, this would be a fruitful direction for future studies.

There was a sizeable drop-off in participants from Phase one to two, which led to a smaller sample size utilized in the current paper. It was therefore not possible to examine differences as a function of cohort type. Also, no data were collected about family relationships, current academic performance of the students, or psychotherapy treatment-seeking behaviors. Furthermore, this study utilized correlation analyses to examine the various relationships with a smaller sample size, which could impact the significance of the relationships. However, we utilized a combination of frequentist and Bayesian approaches when conducting the correlation analyses, which enabled the strengthening of inferences through triangulation (Munafò & Davey Smith, 2018).

## 5. Conclusions

The association between creativity and schizotypy has a long-standing history that continues to be a key area of research interest. Schizotypal features have been linked to the creative process and wellbeing. However, there is a dearth of work on the relationships between schizotypy, wellbeing, and creativity in adolescents, which is consistent with broader schizotypy studies as fewer works have focused on children or adolescents (Raine et al., 2021). In this paper, we investigated the associations between these variables. The relationship between creativity and schizotypy appears to be driven mainly by disorganized features. Conversely, interpersonal schizotypal traits displayed the strongest relationships with measures of wellbeing. Even though overall schizotypy was related to creative potential and wellbeing, diverging subdimensions of schizotypy appear to drive these relationships. Future research can explore how the combination of these schizotypal features can be used to understand and predict measures of creative potential and wellbeing.

## Figures and Tables

**Table 1:** Summary of Sample Characteristics by Cohort.

	All	Cohort 1	Cohort 2	Cohort 3
n	76	38	20	18
Age [Mean, (SD)]	18.34 (1.83)	16.76 (0.28)	18.82 (0.39)	21.15 (0.44)
Age Range	16–22	16–17	18–19	20–22
Gender [n]				
Female	68	34	17	17
Male	7	3	3	1
Non-binary	1	1		

**Table 2:** Descriptive Statistics for Creativity, Wellbeing, and Schizotypy.

Variable	N	Mean	SD
Creativity variables			
AUT fluency	76	4.91	1.54
AUT overall originality	76	4.95	1.43
AUT peak originality	76	5.21	3.08
OKC raw scores	76	1.59	0.94
Creative hobbies	76	6.99	6.67
Wellbeing variables			
Life satisfaction	76	3.72	0.81
Positive affect	72	22.04	3.55
Negative affect	72	16.90	4.30
Mental health	75	2.91	0.72
Schizotypy variables			
SPQ total	72	91.49	18.45
SPQ cognitive perceptual	72	36.01	8.79
SPQ interpersonal	72	14.81	5.30
SPQ disorganized	72	26.74	6.06

*Note:* AUT: Alternate Uses Task, OKC: Overcoming Knowledge Constraints, SPQ: Schizotypal Personality Questionnaire (Brief Revised Updated).

**Table 3:** Correlations Among Creativity, Wellbeing, and Schizotypy Variables.

	Variable		1	2		3		4		5		6		7		8		9		10		11		12		13	
1	AUT fluency	*p*	1	**0.320**	0.005	**0.704**	<0.001	0.076	0.081	−0.111	0.341	0.066	0.582	0.228	0.054	−0.043	0.715	**0.299**	0.009	0.228	0.051	0.125	0.273	**0.290**	0.013	**0.298**	0.011
		BF_10_			7.13		>100		0.18		0.22		0.17		0.90		0.15		4.22		0.95		0.25		3.05		3.58
2	AUT overall originality	*p*	76	1		**0.709**	<0.001	0.086	0.186	0.206	0.075	0.146	0.221	0.133	0.265	0.212	0.068	0.062	0.594	−0.043	0.715	−0.073	0.540	−0.046	0.700	−0.067	0.576
		BF_10_					>100		0.19		0.68		0.31		0.27		0.74		0.17		0.16		0.17		0.16		0.17
3	AUT peak originality	*p*	76	76		1		0.062	0.175	0.107	0.107	0.144	0.227	0.257	**0.029**	0.120	0.306	0.221	0.055	0.040	0.736	−0.078	0.523	0.152	0.200	0.064	0.591
		BF_10_							0.17		0.22		0.30		1.51		0.24		0.87		0.15		0.18		0.330		0.17
4	OKC raw score	*p*	76	76		76		1		0.040	0.733	0.026	0.828	−0.069	0.566	−0.101	0.388	0.097	0.403	0.040	0.904	−0.000	0.529	−0.000	0.890	−0.008	0.950
		BF_10_									0.15		0.15		0.17		0.21		0.20		0.15		0.14		0.15		0.15
5	Life satisfaction	*p*	76	76		76		76		1		**0.613**	<0.001	**−0.334**	0.004	**0.554**	<0.001	0.140	0.229	−0.227	0.052	**−0.400**	<0.001	−0.260	0.027	**−0.387**	0.001
		BF_10_											>100		8.23		>100		0.29		0.93		67.12		1.63		36.65
6	Positive affect	*p*	72	72		72		72		72		1		**−0.327**	0.005	**0.545**	<0.001	0.163	0.171	−0.042	0.731	**−0.386**	0.001	−0.001	0.996	−0.124	0.310
		BF_10_													6.93		<0.001		0.37		0.16		32.85		0.15		0.24
7	Negative affect	*p*	72	72		72		72		72		72		1		**−0.400**	0.001	0.152	0.201	**0.552**	<0.001	**0.303**	0.010	**0.295**	0.013	**0.490**	<0.000
		BF_10_															50.77		0.33		>100		3.73		3.02		>100
8	Mental health	*p*	75	75		75		75		75		71		71		1		0.067	0.566	−0.265	0.023	**−0.450**	<0.001	−0.274	0.020	**−0.406**	<0.000
		BF_10_																	0.17		1.85		>100		2.12		68.08
9	Creative hobbies	*p*	76	76		76		76		76		72		72		75		1		0.198	0.091	0.177	0.128	**0.333**	0.004	0.238	**0.045**
		BF_10_																			0.43		0.32		3.77		1.06
10	SPQ cognitive perceptual	*p*	74	74		74		74		74		70		70		72		74		1		**0.362**	0.002	**0.556**	<0.001	**0.852**	<0.001
		BF_10_																					18.95		>100		>100
11	SPQ interpersonal	*p*	75	75		75		75		75		71		71		74		75		73		1		**0.362**	0.002	**0.673**	<0.001
		BF_10_																							18.67		>100
12	SPQ disorganized	*p*	73	73		73		73		73		70		70		72		73		72		73		1		**0.793**	<0.001
		BF_10_																									>100
13	SPQ total		72	72		72		72		72		76		69		69		72		72		72		72		1	

*Note:* Correlations are shown above the diagonal and *n* below the diagonal. Pearson’s *r* is reported, with the *p* values from the frequentist correlations, and the Bayes factors (BF_10_) from the Bayesian correlations. Only correlations with *p* < 0.05 and BF_10_ > 3 are indicated in bold font. For fields where *p*-values alone are indicated in bold, this reflects partial findings (i.e., where only *p* < 0.05, but BF_10_ < 3). AUT: Alternate Uses Task, OKC: Overcoming Knowledge Constraints, SPQ: Schizotypal Personality Questionnaire (Brief Revised Updated). Background color is used so that the readers are able to locate the values for each row with ease.

**Table 4:** Partial Correlations of Variables with Age as a Covariate.

Variable 1	Variable 2	Pearson’s *r*	*p*-Value
AUT fluency	AUT overall orig.	0.405	<0.001
AUT fluency	AUT peak orig.	0.718	<0.001
AUT overall orig.	AUT peak orig.	0.747	<0.001
Life satisfaction	Positive affect	0.611	<0.001
Life satisfaction	Negative affect	−0.345	0.004
Life satisfaction	Mental health	−0.523	<0.001
Mental health	Positive affect	0.536	<0.001
Mental health	Negative affect	−0.396	0.001
Positive affect	Negative affect	−0.338	0.005
Creative hobbies	AUT fluency	0.284	0.020
SPQ cog. percep.	Negative affect	0.535	<0.001
SPQ interpersonal	Life satisfaction	−0.371	0.002
SPQ interpersonal	Positive affect	0.370	0.002
SPQ interpersonal	Negative affect	0.313	0.010
SPQ interpersonal	Mental health	−0.445	<0.001
SPQ interpersonal	SPQ cog. percep.	0.398	0.001
SPQ disorganized	AUT fluency	0.286	0.019
SPQ disorganized	Negative affect	0.259	0.034
SPQ disorganized	Creative hobbies	0.254	0.038
SPQ disorganized	SPQ cog. percep.	0.514	<0.001
SPQ disorganized	SPQ interpersonal	0.358	0.003
SPQ total	AUT fluency	0.293	0.016
SPQ total	Life satisfaction	−0.354	0.003
SPQ total	Negative affect	0.473	<0.001
SPQ total	Mental health	−0.376	0.002
SPQ total	SPQ cog. percep.	0.841	<0.001
SPQ total	SPQ interpersonal	0.676	<0.001

*Note.* All partial correlations are reported using Pearson’s *r*, with the *p* values from the frequentist methods (n = 68). Variable relationships were assessed for partial correlations if the criteria, *p* < 0.05 and BF_10_ > 3, was met for Frequentist and Bayesian correlations methods (see Table 3). AUT: alternate uses task, cog. percep.: cognitive perceptual, OKC: Overcoming knowledge constraints, orig.: originality; SPQ: Schizotypal Personality Questionnaire (Brief Revised Updated).

## Data Availability

The raw data supporting the conclusions of this article will be made available by the authors on request.

## Note

1 Following the proposals made by Wetzels et al. (2015) based on Jeffreys (1961), the Bayesian findings were interpreted as follows:

(I) Clear evidence for the alternate hypothesis (extremely strong evidence: BF_10_ > 100; very strong evidence: 30–100; strong evidence: 10–30; moderate evidence: 3–10).
(II) Anecdotal evidence for the alternate hypothesis: BF_10_ = 1–3.
(III) No evidence: BF_10_ = 1.
(IV) Anecdotal evidence for the null hypothesis: BF_10_ = 1/3–1.
(V) Clear evidence for the null hypothesis (moderate evidence: BF_10_ = 1/10–1/3; strong evidence: BF_10_ = 1/30–1/10; very strong evidence: BF_10_ = 1/100–1/30; extremely strong evidence: <1/100).

## References

[B1-behavsci-15-00553] Abbott G. R., Do M., Byrne L. K. (2012). Diminished subjective wellbeing in schizotypy is more than just negative affect. Personality and Individual Differences.

[B2-behavsci-15-00553] Abraham A. (2024). The creative brain.

[B3-behavsci-15-00553] Abraham A. (2025). Why the standard definition of creativity fails to capture the creative act. Theory & Psychology.

[B4-behavsci-15-00553] Abraham A., Asquith S., Ahmed H., Bourisly A. K. (2019). Comparing the efficacy of four brief inductions in boosting short-term creativity. Journal of Cognitive Enhancement.

[B5-behavsci-15-00553] Abraham A., Windmann S. (2007). Creative cognition: The diverse operations and the prospect of applying a cognitive neuroscience perspective. Methods.

[B6-behavsci-15-00553] Abraham A., Windmann S. (2008). Selective information processing advantages in creative cognition as a function of schizotypy. Creativity Research Journal.

[B7-behavsci-15-00553] Abu-Akel A., Baxendale L., Mohr C., Sullivan S. (2018). The association between schizotypal traits and social functioning in adolescents from the general population. Psychiatry Research.

[B8-behavsci-15-00553] Acar S., Chen X., Cayirdag N. (2018). Schizophrenia and creativity: A meta-analytic review. Schizophrenia Research.

[B9-behavsci-15-00553] Acar S., Sen S. (2013). A multilevel meta-analysis of the relationship between creativity and schizotypy. Psychology of Aesthetics, Creativity, and the Arts.

[B10-behavsci-15-00553] Asquith S. L., Wang X., Quintana D. S., Abraham A. (2022a). Predictors of creativity in young people: Using frequentist and Bayesian approaches in estimating the importance of individual and contextual factors. Psychology of Aesthetics, Creativity, and the Arts.

[B11-behavsci-15-00553] Asquith S. L., Wang X., Quintana D. S., Abraham A. (2022b). The role of personality traits and leisure activities in predicting wellbeing in young people. BMC Psychology.

[B12-behavsci-15-00553] Asquith S. L., Wang X., Quintana D. S., Abraham A. (2024). Predictors of change in creative thinking abilities in young people: A longitudinal study. The Journal of Creative Behavior.

[B13-behavsci-15-00553] Batey M., Furnham A. (2008). The relationship between measures of creativity and schizotypy. Personality and Individual Differences.

[B14-behavsci-15-00553] Benedek M., Karstendiek M., Ceh S. M., Grabner R. H., Krammer G., Lebuda I., Silvia P. J., Cotter K. N., Li Y., Hu W., Martskvishvili K., Kaufman J. C. (2021). Creativity myths: Prevalence and correlates of misconceptions on creativity. Personality and Individual Differences.

[B15-behavsci-15-00553] Burch G. S. J., Pavelis C., Hemsley D. R., Corr P. J. (2006). Schizotypy and creativity in visual artists. British Journal of Psychology.

[B16-behavsci-15-00553] Christensen R., Haenschel C., Gaigg S. B., Fett A.-K. J. (2022). Loneliness, positive, negative and disorganised Schizotypy before and during the COVID-19 pandemic. Schizophrenia Research: Cognition.

[B17-behavsci-15-00553] Claridge G., Blakey S. (2009). Schizotypy and affective temperament: Relationships with divergent thinking and creativity styles. Personality and Individual Differences.

[B18-behavsci-15-00553] Davidson C. A., Hoffman L., Spaulding W. D. (2016). Schizotypal personality questionnaire—Brief revised (updated): An update of norms, factor structure, and item content in a large non-clinical young adult sample. Psychiatry Research.

[B19-behavsci-15-00553] Debbané M., Vrtička P., Lazouret M., Badoud D., Sander D., Eliez S. (2014). Self-reflection and positive schizotypy in the adolescent brain. Schizophrenia Research.

[B20-behavsci-15-00553] Diener E., Wirtz D., Tov W., Kim-Prieto C., Choi D., Oishi S., Biswas-Diener R. (2010). New well-being measures: Short scales to assess flourishing and positive and negative feelings. Social Indicators Research.

[B21-behavsci-15-00553] Dizinger J. M. B., Doll C. M., Rosen M., Gruen M., Daum L., Schultze-Lutter F., Betz L., Kambeitz J., Vogeley K., Haidl T. K. (2022). Does childhood trauma predict schizotypal traits? A path modelling approach in a cohort of help-seeking subjects. European Archives of Psychiatry and Clinical Neuroscience.

[B22-behavsci-15-00553] Eysenck H. (1995). Genius: The natural history of creativity.

[B23-behavsci-15-00553] Fonseca-Pedrero E., Lemos-Giráldez S., Paino M., Muñiz J. (2011). Schizotypy, emotional–behavioural problems and personality disorder traits in a non-clinical adolescent population. Psychiatry Research.

[B24-behavsci-15-00553] Fumero A., Marrero R., Fonseca-Pedrero E. (2018). Well-being in schizotypy: The effect of subclinical psychotic experiences. Psicothema.

[B25-behavsci-15-00553] Gadermann A. M., Schonert-Reichl K. A., Zumbo B. D. (2010). Investigating validity evidence of the satisfaction with life scale adapted for children. Social Indicators Research.

[B26-behavsci-15-00553] Guilford J. P., Christensen P. R., Merrifeld P. R., Wilson R. C. (1960). Alternate uses manual.

[B27-behavsci-15-00553] Hernández L. M., Kemp K. C., Barrantes-Vidal N., Kwapil T. R. (2023). Disorganized schizotypy and neuroticism in daily life: Examining their overlap and differentiation. Journal of Research in Personality.

[B28-behavsci-15-00553] Jacquet J., Delpech L., Bronchain J., Raynal P. (2020). Creative competencies and cognitive processes associated with creativity are linked with positive schizotypy. Creativity Research Journal.

[B29-behavsci-15-00553] JASP Team (2024). JASP *(Version 0.19.1) [Computer software]*.

[B30-behavsci-15-00553] Jeffreys H. (1961). Theory of probability.

[B31-behavsci-15-00553] Jovanović V. (2015). Beyond the PANAS: Incremental validity of the Scale of Positive and Negative Experience (SPANE) in relation to well-being. Personality and Individual Differences.

[B32-behavsci-15-00553] Karimi Z., Windmann S., Güntürkün O., Abraham A. (2007). Insight problem solving in individuals with high versus low schizotypy. Journal of Research in Personality.

[B33-behavsci-15-00553] Kassambara A. (2023). ggpubr: “ggplot2” based publication ready plots *(Version 0.6.0) [R Package computer software]*.

[B34-behavsci-15-00553] Keyes C. L. M., Annas J. (2009). Feeling good and functioning well: Distinctive concepts in ancient philosophy and contemporary science. The Journal of Positive Psychology.

[B35-behavsci-15-00553] Kwapil T. R., Barrantes-Vidal N. (2015). Schizotypy: Looking back and moving forward. Schizophrenia Bulletin.

[B36-behavsci-15-00553] Lenzenweger M. F. (2018). Schizotypy, schizotypic psychopathology and schizophrenia. World Psychiatry.

[B37-behavsci-15-00553] Liu J., Wong K. K.-Y., Dong F., Raine A., Tuvblad C. (2019). The Schizotypal Personality Questionnaire-Child (SPQ-C): Psychometric properties and relations to behavioral problems with multi-informant ratings. Psychiatry Research.

[B38-behavsci-15-00553] Mason O., Claridge G. (2006). The Oxford-Liverpool Inventory of Feelings and Experiences (O-LIFE): Further description and extended norms. Schizophrenia Research.

[B39-behavsci-15-00553] Meehl P. E. (1962). Schizotaxia, schizotypy, schizophrenia. American Psychologist.

[B40-behavsci-15-00553] Mohr C., Claridge G. (2015). Schizotypy—Do not worry, it is not all worrisome. Schizophrenia Bulletin.

[B41-behavsci-15-00553] Munafò M. R., Davey Smith G. (2018). Robust research needs many lines of evidence. Nature.

[B42-behavsci-15-00553] Oezgen M., Grant P. (2018). Odd and disorganized—Comparing the factor structure of the three major schizotypy inventories. Psychiatry Research.

[B43-behavsci-15-00553] O’Hare K., Watkeys O., Dean K., Laurens K. R., Tzoumakis S., Harris F., Carr V. J., Green M. J. (2023a). Childhood schizotypy and adolescent mental disorder. Schizophrenia Bulletin.

[B44-behavsci-15-00553] O’Hare K., Watkeys O., Whitten T., Dean K., Laurens K. R., Tzoumakis S., Harris F., Carr V. J., Green M. J. (2023b). Cumulative environmental risk in early life: Associations with schizotypy in childhood. Schizophrenia Bulletin.

[B45-behavsci-15-00553] Polner B., Hupuczi E., Kéri S., Kállai J. (2021). Adaptive and maladaptive features of schizotypy clusters in a community sample. Scientific Reports.

[B46-behavsci-15-00553] Quintana D. S., Williams D. R. (2018). Bayesian alternatives for common null-hypothesis significance tests in psychiatry: A non-technical guide using JASP. BMC Psychiatry.

[B48-behavsci-15-00553] Raine A. (1991). The SPQ: A scale for the assessment of schizotypal personality based on DSM-III-R criteria. Schizophrenia Bulletin.

[B49-behavsci-15-00553] Raine A., Wong K. K.-Y., Liu J. (2021). The Schizotypal Personality Questionnaire for Children (SPQ-C): Factor structure, child abuse, and family history of schizotypy. Schizophrenia Bulletin.

[B47-behavsci-15-00553] R Core Team (2022). R: The R project for statistical computing *(Version 4.2.2) [Computer software]*.

[B50-behavsci-15-00553] Rominger C., Fink A., Weiss E. M., Bosch J., Papousek I. (2017). Allusive thinking (remote associations) and auditory top-down inhibition skills differentially predict creativity and positive schizotypy. Cognitive Neuropsychiatry.

[B51-behavsci-15-00553] Runco M. A., Jaeger G. J. (2012). The standard definition of creativity. Creativity Research Journal.

[B52-behavsci-15-00553] Runco M. A., Okuda S. M., Thurston B. J. (1987). The psychometric properties of four systems for scoring divergent thinking tests. Journal of Psychoeducational Assessment.

[B53-behavsci-15-00553] Smith S. M., Ward T. B., Schumacher J. S. (1993). Constraining effects of examples in a creative generation task. Memory & Cognition.

[B54-behavsci-15-00553] Tabak N. T., Weisman de Mamani A. G. (2013). Latent profile analysis of healthy schizotypy within the extended psychosis phenotype. Psychiatry Research.

[B55-behavsci-15-00553] Taylor C. L. (2017). Creativity and mood disorder: A systematic review and meta-analysis. Perspectives on Psychological Science.

[B56-behavsci-15-00553] Toutountzidis D., Gale T. M., Irvine K., Sharma S., Laws K. R. (2022). Childhood trauma and schizotypy in non-clinical samples: A systematic review and meta-analysis. PLoS ONE.

[B57-behavsci-15-00553] Velikonja T., Velthorst E., McClure M. M., Rutter S., Calabrese W. R., Rosell D., Koenigsberg H. W., Goodman M., New A. S., Hazlett E. A., Perez-Rodriguez M. M. (2019). Severe childhood trauma and clinical and neurocognitive features in schizotypal personality disorder. Acta Psychiatrica Scandinavica.

[B58-behavsci-15-00553] Wagenmakers E.-J., Marsman M., Jamil T., Ly A., Verhagen J., Love J., Selker R., Gronau Q. F., Šmíra M., Epskamp S., Matzke D., Rouder J. N., Morey R. D. (2018). Bayesian inference for psychology. Part I: Theoretical advantages and practical ramifications. Psychonomic Bulletin & Review.

[B59-behavsci-15-00553] Wang L., Long H., Plucker J. A., Wang Q., Xu X., Pang W. (2018). High schizotypal individuals are more creative? The mediation roles of overinclusive thinking and cognitive inhibition. Frontiers in Psychology.

[B60-behavsci-15-00553] Wetzels R., van Ravenzwaaij D., Wagenmakers E.-J. (2015). Bayesian analysis. The encyclopedia of clinical psychology.

[B61-behavsci-15-00553] Wickham H., Chang W., Henry L., Pedersen T. L., Takahashi K., Wilke C., Woo K., Yutani H., Dunnington D., van den Brand T., Posit, PBC (2024). ggplot2: Create elegant data visualisations using the grammar of graphics *(Version 3.5.1) [R package computer software]*.

[B62-behavsci-15-00553] Zhang L., Zhao N., Zhu M., Tang M., Liu W., Hong W. (2023). Adverse childhood experiences in patients with schizophrenia: Related factors and clinical implications. Frontiers in Psychiatry.

